# Drug‐induced vasculitis: Thiazide or the COVID‐19 vaccine, which one is guilty? A case report and literature review

**DOI:** 10.1002/ccr3.5978

**Published:** 2022-06-13

**Authors:** Manoochehr Hekmat, Sepideh Jafari Naeini, Zahra Abbasi, Sahar Dadkhahfar

**Affiliations:** ^1^ Department of cardiovascular surgery, Shahid Modarres Hospital Shahid Beheshti university of medical sciences Tehran Iran; ^2^ Cardiovascular research center Shahid Beheshti university of medical sciences Tehran Iran; ^3^ Department of Internal medicine, Shahid Modarres Hospital Shahid Beheshti university of medical sciences Tehran Iran; ^4^ Skin research center Shahid Beheshti university of medical sciences Tehran Iran

**Keywords:** COVID‐19 vaccines, thiazides, vasculitis, warfarin

## Abstract

A middle‐aged woman with a history of COVID‐19 vaccine administration and valve replacement surgery was admitted with bilateral palpable purpuric lesions in the lower extremities and headache. Based on the initial diagnosis of vasculitis, corticosteroid therapy was initiated, which led to the resolution of skin lesions.

## Graphical abstract

Drug‐induced vasculitis should be considered in the setting of vasculitis after the initiation of a new medication or the administration of COVID‐19 vaccines.

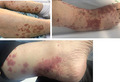

## 1 INTRODUCTION

Vasculitis is specified by the inflammation of the blood vessel wall and can affect any organ system of the body. Cutaneous vasculitis may be^1^ a skin‐limited disease^2^; a primary cutaneous vasculitis with secondary systemic involvement^3^; or a systemic vasculitis with cutaneous manifestations. Cutaneous findings of vasculitis reflect the size of the vessels involved

As a prevalent non‐infectious cause of small cutaneous vasculitis, drug‐induced vasculitis (DIV) can present with a broad spectrum of clinical signs and symptoms. As an inflammatory process in blood vessels, it may affect different organs with localized or systemic manifestations. Initial diagnosis should be made after the exclusion of other causes of vasculitis, such as infectious diseases (especially viral and parasitic infections), autoimmune collagen vascular diseases, and different related neoplasms.^1^, ^2^, ^3^ Here,we present a case of drug‐induced vasculitis with a specific initial presentation and course.

## 2 CASE PRESENTATION

**FIGURE 1:**
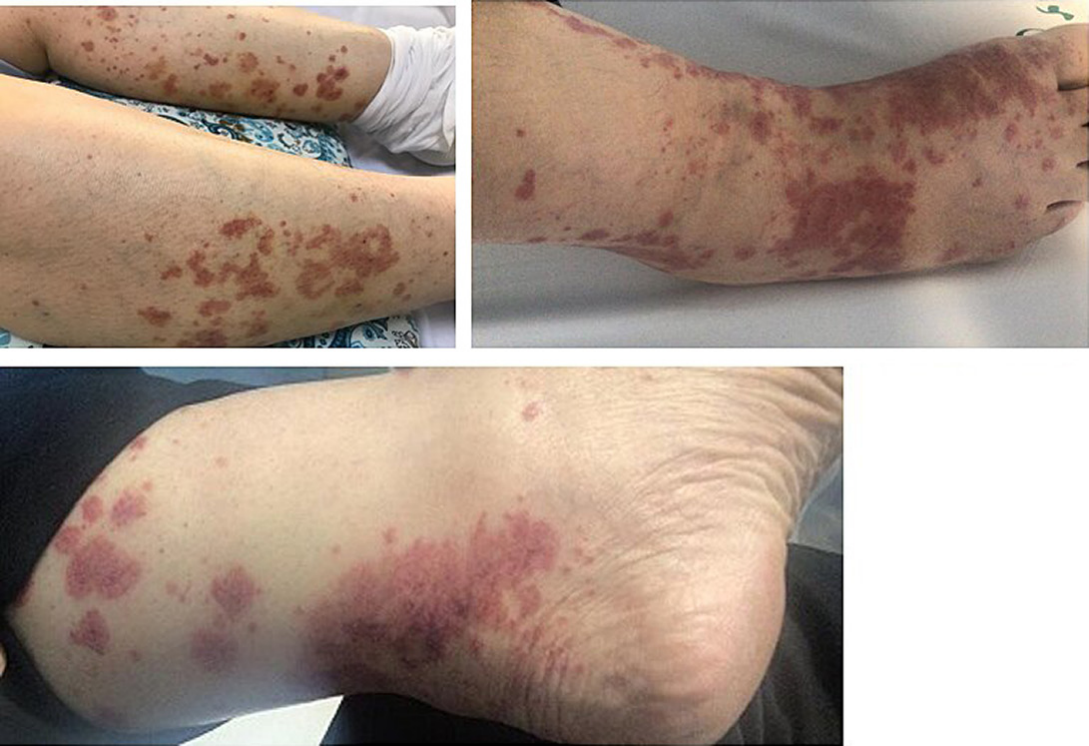
Palpable purpuric lesions in both lower extremities with hemorrhagic changes

A 55‐year‐old woman was admitted with progressive itching, palpable purpura in both lower extremities and pruritus without systemic symptoms, such as arthralgia, myalgia, and weight loss or fever (Figure 1). Her symptoms had started 3 days before admission, and some of her lesions were hemorrhagic. Concomitantly, she had complained of a vague headache during the days prior to being hospitalized. She had a history of combined mitral and aortic valve replacement (mechanical valve) 1 week prior to her admission due to rheumatismal heart disease and a history of COVID‐19 vaccination (Sinopharm BIBP COVID‐19 vaccine) 3 weeks prior to the onset of symptoms. She also had a history of hypothyroidism treated with levothyroxine and was discharged after cardiac surgery with warfarin, hydrochlorthiazide (HCTZ) (25 mg once daily), propranolol (10 mg BID), levothyroxine (100 microgram per day), and pantoprazole (20 mg daily). She also denied any allergies to medications or vaccines or any history of dermatologic problems. There was no family history of rheumatologic or dermatologic problems.

On admission, she was completely alert with stable vital signs (blood pressure = 120/75 mmHg, pulse rate = 80 /minute, respiratory rate = 14 per minute without fever. Laboratory tests revealed creatinine =1.4 mg/dL, total bilirubin = 1.6 mg/ dl, direct bilirubin =0.2 mg/ dl, white blood cells = 7300/cm3, International normalized ratio (INR) =3.7,negative serology, and polymerase chain reaction (PCR) for COVID‐19 infection, qualitative C‐reactive protein (CRP) =2+, and thyroid stimulating hormone (TSH) = 16.4 mIU/ml. All the rheumatologic tests, including antinuclear antibody (ANA), antineutrophil cytoplasmic antibodies (ANCA), rheumatoid factor (RF), anti RO, anti‐double‐stranded DNA (anti ds‐DNA) and Anti‐CCPs (cyclic interlineated peptides), hepatitis panel, and human immunodeficiency virus antibody (HIV Ab) were negative and complement levels were normal.

**FIGURE 2:**
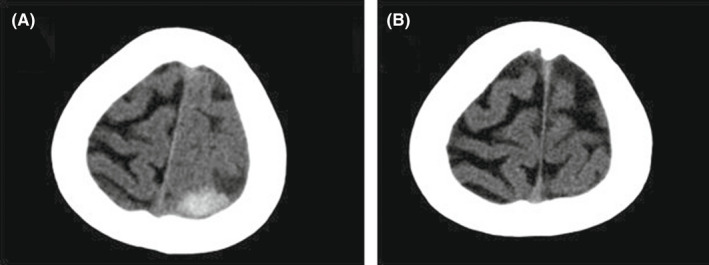
(A) Spiral brain computed tomography (CT) showed evidence of acute and subacute foci of For Review Only subdural hematoma adjacent to the left temporal lobe. (B) The hemorrhagic lesion disappeared after 1 month

Echocardiography demonstrated normal biventricular size and function with a large pericardial effusion (about 30 mm) posterior to the left ventricle without a compressive effect. A spiral brain computed tomography (CT) scan was performed, which showed evidence of acute and subacute foci of subdural hematoma adjacent to the left temporal lobe (Figure 2).

As a result, we had to transiently hold the administration of the anticoagulant regimen until the next CT scans (performed after 48 hours and again after 5 days) confirmed the stabilization of the hemorrhagic area without expansion.

**FIGURE 3:**
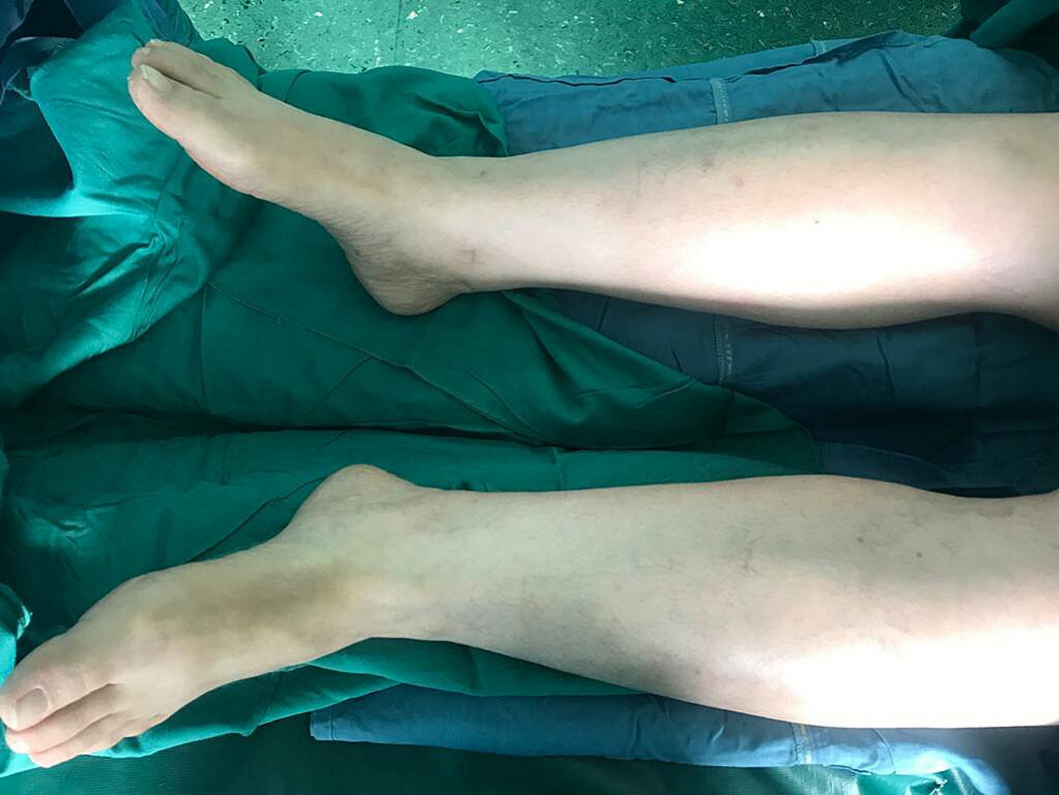
Cutaneous lesions disappeared gradually after the initiation of corticosteroid

Rheumatology and dermatology consultations were requested, and the initiation of corticosteroid therapy was recommended based on the diagnosis of vasculitis. The patient refused to undergo a skin biopsy and methylprenosiolone 1 gr/ day was initiated based on the clinical scenario, with the probable diagnosis of vasculitis, which led to rapid improvement of the lesions (Figure 3). She was discharged from the hospital with oral prednisolon, which was continued and tapered over the next 2 months and colchicin 1 mg /d was initiated and continued for 2 months. The patient did not show any complications and the follow‐up brain CT showed elimination of the hemorrhagic focus after about 1 month. She received a second dose of sinopharm 2 months after the first dose without any complications. Three months later, she underwent drainage of a massive pericardial effusion which had been reported early after surgery but had not shown any reduction in size during the post‐operative period.

## 3 DISCUSSION

Leukocytoklastic vasculitis (small vessel vasculitis with neutrophil infiltration) due to different medications has been reported to be the cause of 1/3 of cases of cutaneous vasculitis.^1^, ^4^, ^5^, ^6^ Drug‐induced vasculitis (DIV) can involve vessels of variable sizes but is less established in large vessels, such as the aorta. Although not very prevalent, concomitant involvement of the coronary or cerebral vasculature has also been reported.^5^ The diagnosis of DIV should be made by exclusion of other probable causes, as mentioned before^1^.

Although warfarin has been considered to be the potential cause of vasculitis, it more commonly causes microvascular occlusion as opposed to leukocytoklastic vasculitis,^7^, ^8^ We were unable to discontinue warfarin in our patient due to the lack of an alternative based on the history of mechanical valve replacement. Absence of the recurrence of symptoms after the discontinuation of corticosteroids made warfarin less likely to be considered the cause of vasculitis because based on previous reports, and contrary to our study, clinical symptoms relapsed in most cases after reinitiating warfarin.^4^ Another potential cause of the occurrence of vasculitis is the use of thiazides, which has been known for many years^1^.Hypersensitivity vasculitis due to HCTZ is a rare but known entity which could not be ruled out in our case. Pathologic findings have a substantial role in eliminating infectious causes or malignancies. They can also determine the presence of immune complexes in the small vessels.^9^ Finally, we were curious whether the administration of COVID‐19 vaccines could result in vasculitis. Although there have recently been some reports about the use of mRNA based COVID‐19 vaccines as a cause of leukocytoclastic vasculitis,^10^, ^11^, ^12^, ^13^, ^14^ the role of inactivated whole virus vaccines such as Sinopharm, Sinovac (CoronaVac), and COVAXIN (Bharat Biotech) should not be overlooked^15^, ^16^, ^17^.

By discontinuing thiazides and receiving low dose of oral prednisolone, our patient showed significant improvement and all the lesions disappeared early after the initiation of corticosteroids. Hemorrhagic stroke resolved gradually without any complications. After a 3‐month follow‐up period, her condition did not recur, and she underwent drainage of a massive pericardial effusion without any complications. Based on the non‐inflammatory pathologic findings, it appeared that the effusion had resulted from surgical intervention.

## 4 CONCLUSION

Irrespective of the low prevalence, drug‐induced vasculitis (DIV) should be considered in the presence of vasculitis of unknown origin. Recently, multiple cases of COVID‐19 vaccine‐induced vasculitis have been reported, which necessitate further evaluation to diagnose of the underlying cause.

## AUTHORS CONTRIBUTIONS

Manoocheh Hekmat involved in data gathering. Sepideh Jafari Naeini involved in data gathering, writing and editing the text. Zahra Abbasi and Sahar Dadkhahfar involved in editing the text.

## CONFLICT OF INTEREST

None declared.

## CONSENT

Written informed consent was obtained from the patient who participated in this study.

## Data Availability

The data that support the findings of this study are available on request from the corresponding author. The data are not publicly available due to privacy or ethical restrictions.
